# Targeting LY6E Inhibits Neuroblastoma Progression and Suppresses M2 Macrophage Polarization

**DOI:** 10.1155/humu/3003097

**Published:** 2026-04-15

**Authors:** Lijuan Li, Yu Zeng, Qinfen Zhang, Gaojian Zhuang, Yu Liu, Xuan Wang, Yuqi Wang

**Affiliations:** ^1^ Health Management Center, Tianjin Medical University Cancer Institute & Hospital, National Clinical Research Center for Cancer, Tianjin′s Clinical Research Center for Cancer, Key Laboratory of Cancer Prevention and Therapy, Tianjin, China, tijmu.edu.cn; ^2^ Department of Immunology, Tianjin Medical University Cancer Institute & Hospital, National Clinical Research Center for Cancer, Tianjin′s Clinical Research Center for Cancer, Key Laboratory of Cancer Immunology and Biotherapy, State Key Laboratory of Druggability Evaluation and Systematic Translational Medicine, Tianjin, China, tijmu.edu.cn; ^3^ Department of Thyroid and Breast Surgery, The Affiliated Qingyuan Hospital (Qingyuan People′s Hospital) of Guangzhou Medical University, Qingyuan, Guangdong, China; ^4^ The Third Department of Breast Cancer, Tianjin Medical University Cancer Institute & Hospital, National Clinical Research Center for Cancer, Tianjin′s Clinical Research Center for Cancer, Key Laboratory of Cancer Prevention and Therapy, Tianjin, China, tijmu.edu.cn; ^5^ Department of Phase I clinical trial, Tianjin Medical University Cancer Institute & Hospital, National Clinical Research Center for Cancer, Tianjin′s Clinical Research Center for Cancer, Key Laboratory of Cancer Prevention and Therapy, Tianjin, China, tijmu.edu.cn

**Keywords:** LY6E, neuroblastoma, prognosis, risk score model, tumor-associated macrophages (TAMs)

## Abstract

Neuroblastoma is a pediatric malignancy characterized by significant clinical heterogeneity. Although MYCN amplification is a well‐established marker of high‐risk disease, its interplay with the tumor immune microenvironment—particularly tumor‐associated macrophages (TAMs)—remains poorly understood. In this study, we developed an integrated gene signature incorporating genes associated with both MYCN amplification status and TAM infiltration, leading to the identification of 16 differentially expressed genes implicated in both biological processes. Six of these genes (CMBL, LY6E, KLRB1, CTSH, CD3D, and PTGDS) were utilized to construct a risk‐scoring model that effectively stratified neuroblastoma patients into high‐ and low‐risk groups with significantly distinct clinical outcomes (*p* < 0.001). Notably, LY6E emerged as the most prognostically significant gene within the signature. More importantly, we revealed that LY6E modulates M2‐type macrophage polarization in neuroblastoma for the first time, suggesting a novel mechanism through which it may contribute to shaping an immunosuppressive tumor microenvironment.

## 1. Introduction

Neuroblastoma (NB) arises from the embryonic peripheral sympathetic nervous system, where neural crest‐derived precursors fail to undergo proper sympathetic neuronal differentiation due to genomic and epigenetic alterations [1]. Representing roughly 10% of childhood malignancies, NB predominantly affects children under 5 years of age [2]. Depending on the patient′s age, stage, histologic features, and genetic alterations, NB may present with spontaneous regression or exhibit highly aggressive and metastatic properties [3]. As a result, there is a huge variation in the 5‐year survival of patients with NB, ranging from 90%–95% for low risk to 40%–50% for high risk. Some high‐risk patients develop chemoresistance and relapse, and their survival rate is below 40% [4].

Among all types of genetic alterations identified in NB, amplification of MYCN accounts for 20% of cases. MYCN amplification (MNA) has long been regarded as a symbol associated with aggressive phenotypes, treatment resistance, and poor outcome [5]. MYCN can promote cancer progression by encoding an E‐box‐binding [6]. However, MYCN is not amenable to direct pharmacological inhibition [5]. Disialoganglioside (GD2) is highly expressed in NB cells but scarcely expressed in normal tissues; therefore, monoclonal antibody (mAb) therapy for GD2 has become a specific targeted therapeutic regimen for high‐risk and relapsed/refractory. Nevertheless, the effectiveness of this therapeutic approach is constrained, accompanied by a substantial incidence of disease recurrence. Therefore, there is an urgent need to find new therapeutic approaches [7].

Given the crucial involvement of tumor microenvironment (TME) architecture and functionality in oncogenesis and metastatic progression, TME‐targeted modulation has emerged as a significant focus within cancer immunotherapeutic strategies. The TME contains multiple cells, such as macrophages, dendritic cells (DCs), fibroblasts, endothelial cells, inflammatory cells, lymphocytes, extracellular matrix (ECM), vasculature, and chemokines. The TME is marked by abnormal changes in tumor vasculature, which can alter the normal microenvironment and contribute to tumor progression and poor treatment effects. Existing research indicates that the TME significantly contributes to the development of chemoresistance and suboptimal therapeutic outcomes. Therefore, modulation of TME is one of the hotspots in cancer immunotherapy [8–10].

As the predominant immune population within the TME, macrophages demonstrate significant phenotypic diversity [11]. Macrophages can exhibit both pro tumorigenic and antitumorigenic abilities [12, 13]. Due to varied cross talk and stimulation of chemokines. Tumor‐associated macrophages (TAMs) can be recruited by multiple TME components, including cytokines, chemokines, and exosomes. Subsequently, these environmental factors could induce TAMs polarization into M1 states that inhibit tumor growth or M2 states that promote tumor growth [14]. The polarization process of TAMs is continuous and gradually moves toward the M2‐like state, accompanied by the progression of tumor malignancy, which constitutes a positive feedback loop accelerating tumor growth and metastasis. Several studies have shown that the immune system plays a key role in the prognosis and treatment of NB [15]. Therefore, studying the various factors and mechanisms affecting TAMs polarization is crucial for developing new strategies for tumor treatment [16]. Notably, although members of the LY6 family (e.g., LY6E and LY6K) have been implicated in tumor progression and immune regulation across multiple malignancies—including gastric carcinoma, pancreatic cancer, and triple‐negative breast cancer—their expression patterns and functional roles in NB remain incompletely uncharacterized [17, 18]. Prior studies on LY6E have focused on its capacity to modulate lymphocyte activation, tumor cell proliferation, and epithelial‐mesenchymal transition [19]. However, no research has addressed whether LY6E is expressed in NB, noLY6E′sit explored LY6E′s potential involvement in shaping the NB TME—particularly its interaction with TAMs, a key determinant of NB aggressiveness. Thus, the present study fills two critical gaps: (1) It is the first to identify LY6E as a MYCN‐associated gene in NB; (2) it uncovers a novel function of LY6E in promoting M2 macrophage polarization, a mechanism distinct from its previously reported immunomodulatory roles.

In this study, we performed differential gene expression analysis on MYCN‐amplified and nonamplified NB samples using the GSE49710 dataset and conducted an intersection analysis with genes related to TAMs. After further screening of the 16 identified MYCN‐TAM‐differentially expressed genes (DEGs), a prognostic risk model was developed utilizing six pivotal genes. Among these, LY6E was identified as a critical target because of its significantly differential expression and strong association with overall survival (OS). Subsequent experimental validation was conducted to elucidate the biological role of LY6E in NB and its function in M2 macrophage polarization. The experimental data demonstrated that LY6E markedly increased the oncogenic capacity of NB cells while inducing macrophage differentiation into the M2 phenotype.

## 2. Materials and Methods

### 2.1. Data Collection and Preprocessing

RNA‐seq data (row count normalized) and associated clinical information for 498 primary NB cases were acquired from the GSE49710 dataset available through the Gene Expression Omnibus (GEO) repository (accession: https://www.ncbi.nlm.nih.govgeo/). We also utilized the GSE62564 dataset for external validation of our signature.

### 2.2. Identification of MYCN–TAM‐DEGs and Biological Functional Annotation

DEGs of the GEO dataset was performed using the limma package in R, applying thresholds of *p* < 0.05 and |logFC| ≥ 0.5 for mRNA screening. Both upregulated and downregulated genes in both datasets were regarded as DEGs [20]. To characterize the polarization status of TAMs, we selected canonical markers for M1 (IL‐1*β*, TNF‐*α*, and CD86) and M2 (TGF‐*β*, CD163, and CD206) macrophages based on published consensus and performed validation assays [21, 22]. As the DEGs identified in this step were associated with both MNA and TAM, they were defined as MYCN–TAM‐DEGs. Furthermore, we used the “clusterProfiler” package to analyze the GO and KEGG pathways that regulate MYCN–TAM‐DEGs both up and down. Statistical significance was defined by an adjusted *p* value threshold of less than 0.05.

### 2.3. Construction and Validation of the Prognostic Model

Univariate Cox proportional hazards regression was first performed to screen for genes significantly associated with OS (*p* < 0.05). This step helps eliminate genes with weak prognostic relevance and reduces noise interference for subsequent LASSO analysis. Univariate and multivariate Cox proportional hazards regression analyzes of the 16 MYCN–TAM‐DEGs identified six genes that were significantly associated with OS (*p* < 0.05). LASSO Cox regression was implemented using the “glmnet” package in R, with lambda values determined via 10‐fold cross‐validation. Lambda.min (the lambda value corresponding to the minimum cross‐validation error) was selected as the optimal parameter for constructing the prognostic model, which ensures the model′s robustness and predictive performance while achieving feature selection. Subsequent LASSO Cox regression analysis of these six genes yielded a prognostic signature consisting of the six genes, formulated as follows: risk score  =  (0.31 × CMBL)  +  (0.21 × LY6E)  −  (0.28 × KLRB1) + (0.14 × CTSH) + (0.17 × CD3D) − (0.14 × PTGDS). Using the median risk score as the cutoff value, patients were stratified into high‐ and low‐risk cohorts, and survival differences between the two cohorts were evaluated using the log‐rank test. The predictive accuracy of the model was assessed via Kaplan–Meier (K‐M) survival curves and quantification of the area under the receiver operating characteristic (ROC) curve (AUC).

### 2.4. Different Clinical Parameters and Correlation Heat Map Analysis

We obtained clinical information from patients with NB, including OS status (dead and alive) and INSS stage (Stages 1, 2, 3, 4, and 4S). Based on clinical risk stratification, the patients were categorized into two groups. The expression differences of the six key DEGs across the different subgroups and the risk score distributions among these groups were analyzed. Furthermore, correlations between the six key DEGs and risk scores with OS and event‐free survival (EFS) were evaluated. The coexpression relationships among the six key DEGs and the risk score were examined using the Spearman correlation coefficient via R software.

### 2.5. Drug Sensitivity Analysis

Drug sensitivity data comprising half‐maximal inhibitory concentration (IC50) values and corresponding transcriptomic profiles were acquired from two pharmacogenomics resources: 265 compounds from the Genomics of Drug Sensitivity in Cancer (GDSC) database and 481 compounds from the Cancer Treatment Response Portal (CTRP) database, covering 1001 cellular models. The relationship between transcriptional expression and drug sensitivity was examined using Pearson′s correlation method. Statistical significance was determined using an FDR threshold of < 0.05, with bubble plots employed to visualize gene‐drug associations.

### 2.6. Cell Culture and Cell Transfection

The study utilized two NB cell lines: SK‐N‐BE(2) with MNA and SH‐SY5Y without MNA. Cells were cultured in DMEM/F12 medium containing 10% fetal bovine serum (FBS) and 1% penicillin‐streptomycin and incubated at 37°C in a 5% CO_2_ humidified atmosphere. THP‐1 cells were cultured in RPMI 1640 medium supplemented with 10% FBS. THP‐1 monocyte differentiation into macrophages was stimulated with 100 ng/ml PMA (MedChemExpress, HY‐18739) for 24–48 h, after which the differentiated macrophages were cocultured for 48 h with siRNA‐transfected SK‐N‐BE(2) and SH‐SY5Y NB cells.

We synthesized siRNA against LY6E (Tsingke Biotechnology Co., Ltd, Beijing, China) to study LY6E function. The sequences of siRNA were listed in Table S1. NB cells were transfected using Lipofectamine 2000 (Invitrogen, Waltham, Massachusettes, United States) according to the manufacturer′s protocol. Following a 6‐h incubation period, the transfection medium without serum was substituted with complete culture medium supplemented with 10% FBS.

### 2.7. Real‐Time Quantitative PCR (qRT‐PCR)

Total RNA isolation was performed with TRIzol reagent (SparkJade, China; Cat.No. AC0101‐B) following the manufacturer′s protocol. Quantitative assessment of mRNA expression was conducted using 2 × HQ SYBR qPCR Mix (ZOMANBIO, Beijing; ZF501) on an Applied Biosystems 7500 Fast Real‐Time PCR platform. Primer sequences employed in this investigation were detailed in Table S2.

### 2.8. Cell Proliferation Assays

Cellular proliferation rates were determined using commercially available CCK‐8 (C6005M; US EverBright, California) and EdU (C6015M; US EverBright) assay kits following the manufacturer′s protocols. In CCK8 proliferation assays, cells were plated in 96‐well plates at a density of 1000 cells per well and maintained at 37°C with 5% CO_2_ supplementation. CCK8 reagent was added for a duration of 2 h to each well, then incubated for 0, 24, 48, and 72. In the EdU proliferation assay, cells were plated in 24‐well plates and subsequently fixed with 4% paraformaldehyde followed by staining after 24 h of culture.

### 2.9. Cell Migration Assay

Cell migratory capacity was evaluated employing Transwell migration assays (Corning, New York), wherein SK‐N‐BE(2) and SH‐SY5Y cells resuspended in serum‐free medium were plated in the upper chamber. The lower chamber of the Transwell system was supplemented with 600 *μ*L of medium containing 15% FBS. After 24 h of incubation at 37°C with 5% CO_2_, cells that had migrated to the lower chamber were fixed with 4% paraformaldehyde and stained with 0.1% crystal violet.

### 2.10. Coculture System

A Transwell coculture system featuring 0.4‐*μ*m porous membranes (Corning, New York, United States) was employed to culture NB cell lines with THP‐1‐derived macrophages in physically separated upper and lower chambers. Transfected NB cells (5 × 10^5^ cells/well) were seeded in upper chambers and maintained in coculture for 72 h with THP‐1‐differentiated macrophages (1 × 10^6^ cells/well) previously plated in lower compartments of 6‐well plates.

### 2.11. Enzyme‐Linked Immunosorbent Assay (ELISA)

Quantification of human interleukin‐10 (IL‐10) and interleukin‐4 (IL‐4) concentrations was performed with commercial ELISA kits (Boster Biological Technology, Wuhan; IL‐4: EK0404, IL‐10: EK0416) according to the manufacturer′s protocols, with optical density measurements conducted at 450 nm.

### 2.12. Statistical Methods

All statistical analyses were performed using R Version 4.2.1. Intergroup comparisons were assessed with two‐tailed unpaired *t*‐tests and Wilcoxon rank‐sum tests. OS was analyzed through K‐M methodology with log‐rank testing. All tests were two‐sided with statistical significance defined as *p* < 0.05. Significance levels are denoted by asterisks ( ^∗^ *p* < 0.05,  ^∗∗^ *p* < 0.01, and  ^∗∗∗^ *p* < 0.001).

## 3. Results

### 3.1. Identification of MYCN‐DEGs and MYCN–TAM‐DEGs

Patients with NB with amplification of the MYCN gene usually exhibit a poor prognosis [23]. We first searched for MYCN‐DEGs. After downloading the GSE49710 dataset, we normalized the microarray data. Differential expression is shown as a volcano plot (Figure 1a). A total of 476 MYCN‐DEGs were identified for further investigation. According to the Venn diagram analysis, we identified 16 overlapping MYCN‐TAM‐DEGs, as shown in Figure 1b. Subsequently, we evaluated the prognostic value of these 16 candidate genes using univariate Cox regression analysis. The results demonstrated that all 16 genes were significantly correlated with the prognosis of NB patients, among which the expression levels of CMBL and LY6E were significantly positively correlated with unfavorable prognosis in NB (Figure 1c). Finally, functional enrichment analysis revealed that these genes were predominantly enriched in the neuroactive ligand‐receptor interaction pathway, while also showing associations with central carbon metabolism and tyrosine metabolic processes (Figures 2a, 2b, and 2c).

Figure 1Screening of candidate genes and construction of signature. (a) Volcano plot displaying MYCN‐DEGs between MYCN‐amplified and nonamplified NB patients in GSE49710 and the red, black, and blue circles indicate upregulated, none significant, and downregulated MYCN genes. (b) Venn diagram illustrating the intersection of MYCN‐DEGs with TAM‐related genes. The orange regions represent 460 MYCN‐DEGs, the blue regions represent 228 TAM‐related genes, and the gray regions represent the overlap of 16 genes. (c) Forest diagram exhibiting the univariate Cox proportional hazard regression for 16 genes, and all candidate genes were associated with OS in GSE49710 datasets.

**Figure:**
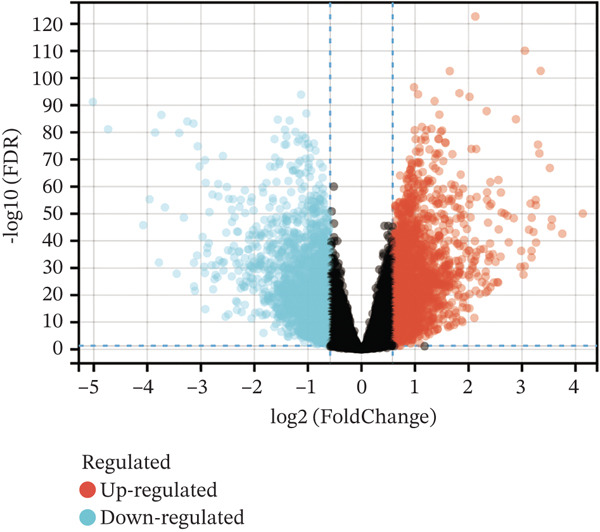
(a)

**Figure:**
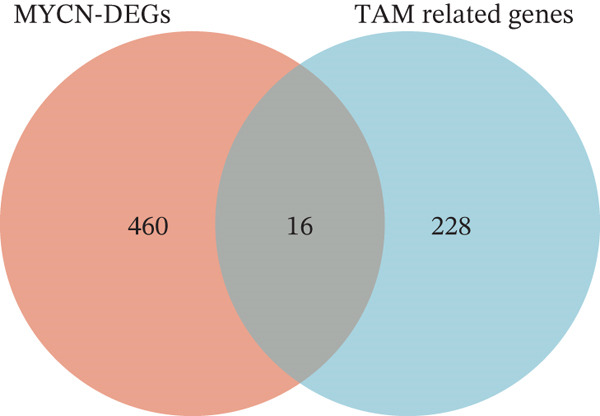
(b)

**Figure:**
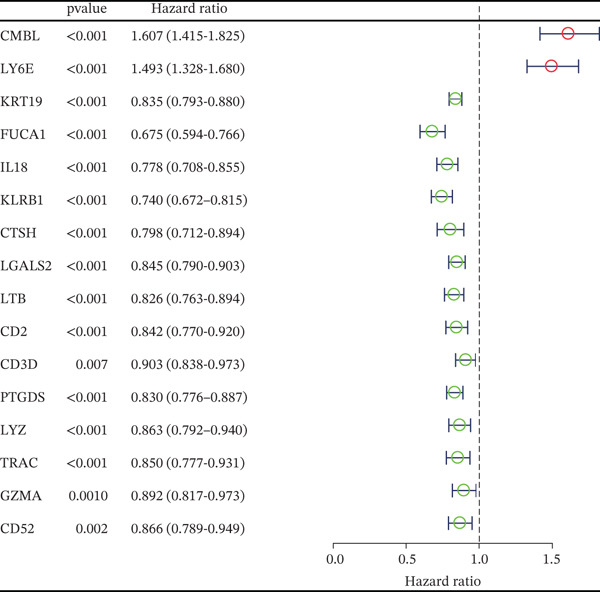
(c)

Figure 2Enrichment analyzes with MYCN‐DEGs and TAMs‐related differentially expressed genes (MYCN‐TAM‐DEGs). (a) Bubble plot of KEGG enrichment pathways analysis. A darker color and a larger bubble denote a more obvious difference. (b) Barplot of KEGG pathway enrichment analysis. (c) The genes are linked to their assigned pathway terms through different colored ribbons and displayed according to the observed log10 *p* value, which is characterized by the intensity of red squares.

**Figure:**
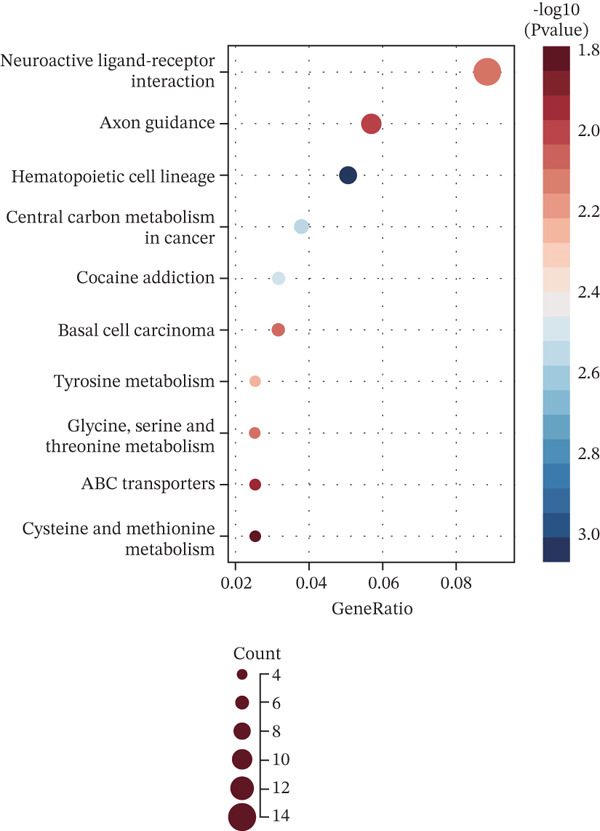
(a)

**Figure:**
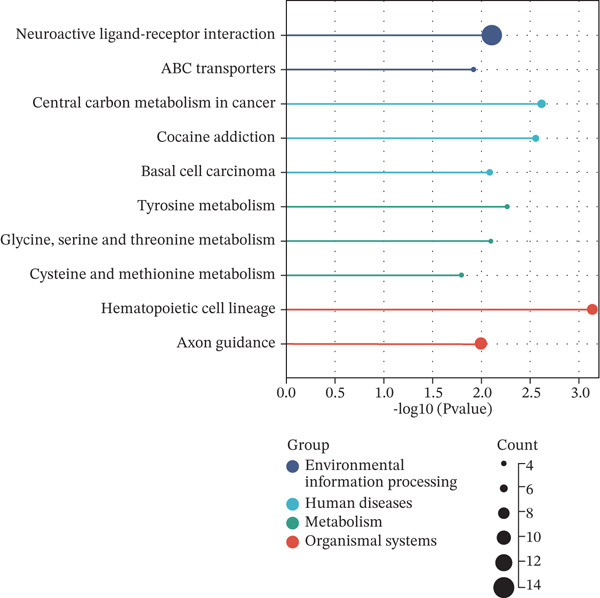
(b)

**Figure:**
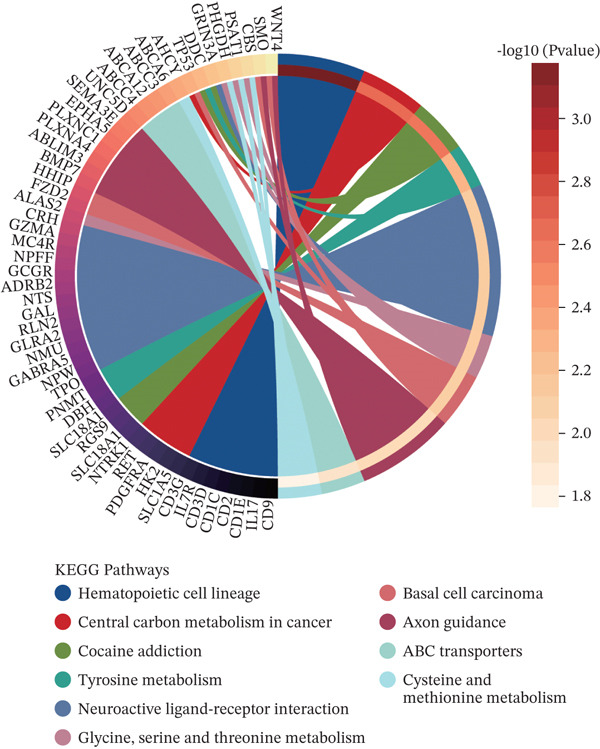
(c)

### 3.2. Development and Validation of the Six‐Gene Prognostic Signature

To construct a robust prognostic signature, the 16 prognostic MYCN–TAM‐DEGs were subjected to multivariate Cox regression analysis, which identified six genes (CMBL, LY6E, KLRB1, CTSH, CD3D, and PTGDS) significantly associated with OS in NB patients (Figure 3a). Subsequently, a LASSO regression model with lambda.min criteria was applied, integrating the expression profiles and regression coefficients of these six genes to establish the prognostic signature. The risk score was calculated as a weighted sum based on their respective coefficients: (0.31 × CMBL) + (0.21 × LY6E) − (0.28 × KLRB1) + (0.14 × CTSH) + (0.17 × CD3D) − (0.14 × PTGDS). The expression patterns and survival outcomes of this six‐gene signature across NB patients are visually summarized in heat maps (Figures 3b, 3c, and 3f), whereas Figure 3d illustrates the quantitative expression trends of each gene. ROC curve analysis demonstrated the strong predictive accuracy of the signature for 1, 3, and 5‐year OS, with AUC values of 0.70, 0.64, and 0.57, respectively (Figure 3e). Consistently, K‐M survival analysis confirmed significantly worse OS in the high‐risk group (Figure 3g). The signature also exhibited reliable predictive performance for EFS, with corresponding AUCs of 0.66, 0.77, and 0.77 at 1, 3, and 5 years (Figure S1). Furthermore, single‐gene prognostic analysis of the six DEGs revealed that high expression of CMBL and LY6E was significantly associated with adverse outcomes (all *p* < 0.001), whereas elevated expression of PTGDS, CD3D, KLRB1, and CTSH correlated with favorable prognosis (all *p* < 0.001) (Figures 4a, 4b, 4c, 4d, 4e, and 4f).

Figure 3Construction of the prognostic risk model. (a) Multivariate Cox regression analysis showed that six genes (CMBL, LY6E, KLRB1, CTSH, CD3D, and PTGDS) correlated with OS in NB patients; the black dashed line represents hazard ratio (HR) = 1. (b) Lasso coefficient profile for prognostic value. (c) Partial likelihood distribution with corresponding *λ*‐logarithm values. (d) Expression trends and amounts of six genes included in the mode. (e) ROC curve for survival prediction. (f) Patients′ risk scores and survival status distributions are displayed along with the expression features of six key DEGs. (g) Survival curves of patients in high RiskScore and low RiskScore.

**Figure:**
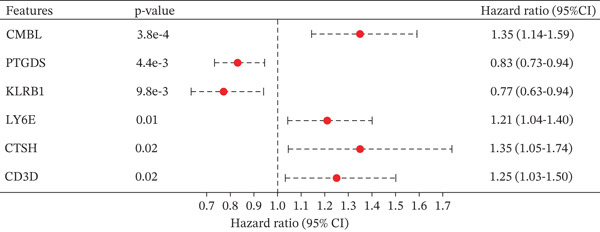
(a)

**Figure:**
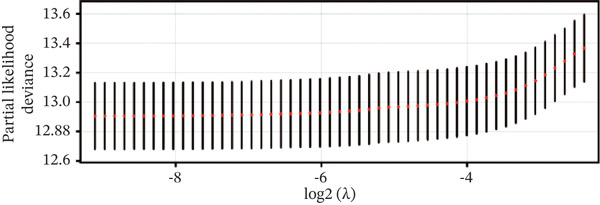
(b)

**Figure:**
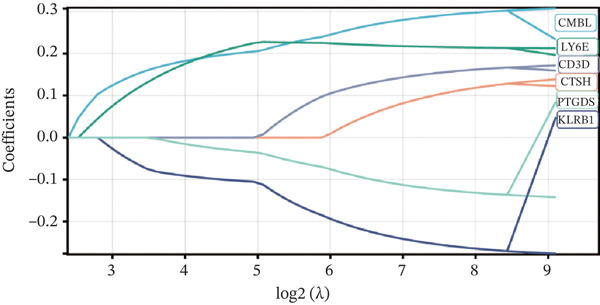
(c)

**Figure:**
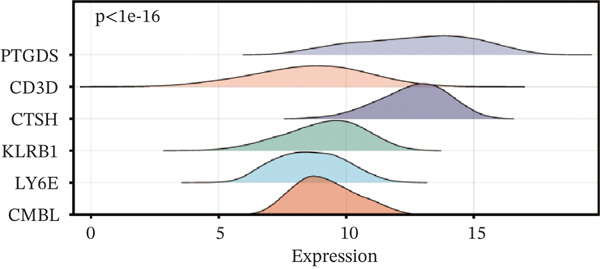
(d)

**Figure:**
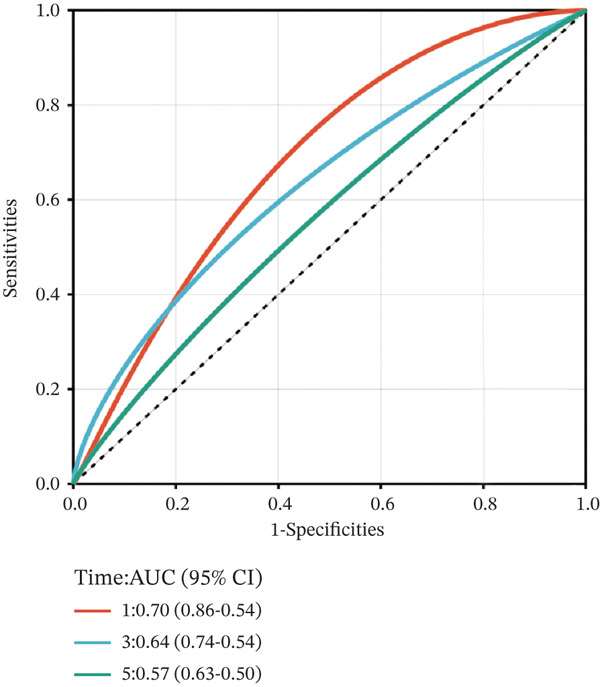
(e)

**Figure:**
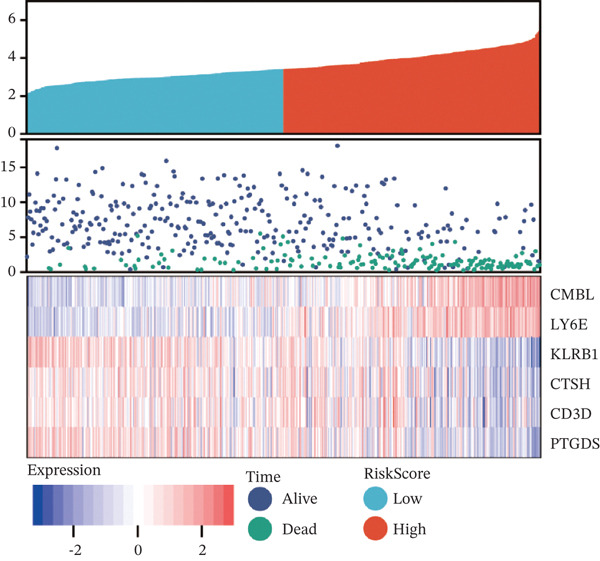
(f)

**Figure:**
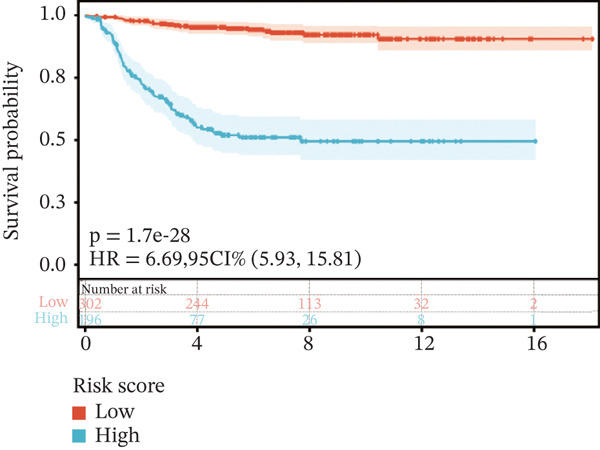
(g)

Figure 4Kaplan‐Meier (K‐M) curve for prognostic prediction in NB. (a–f) Survival curves of the relationship between CMBL, PTGDS, CD3D, KLRB1, LY6E, and CTSH genes and the prognosis of NB patients, respectively. The blue curve indicates high gene expression, whereas the red curve indicates low gene expression.

**Figure:**
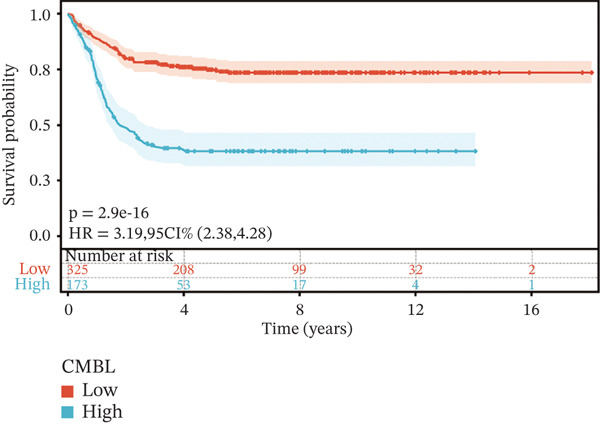
(a)

**Figure:**
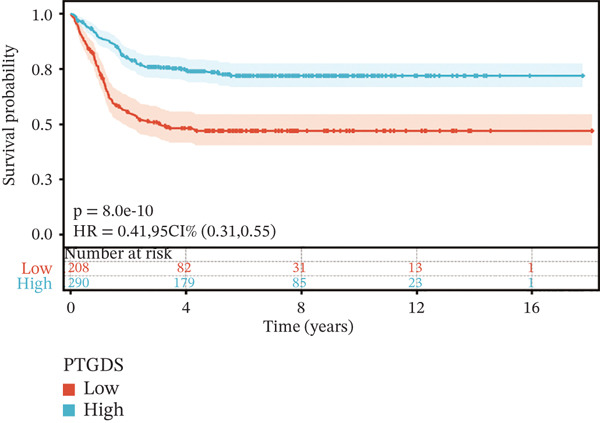
(b)

**Figure:**
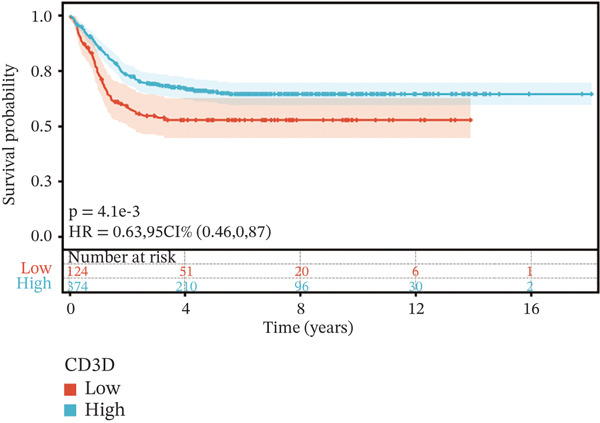
(c)

**Figure:**
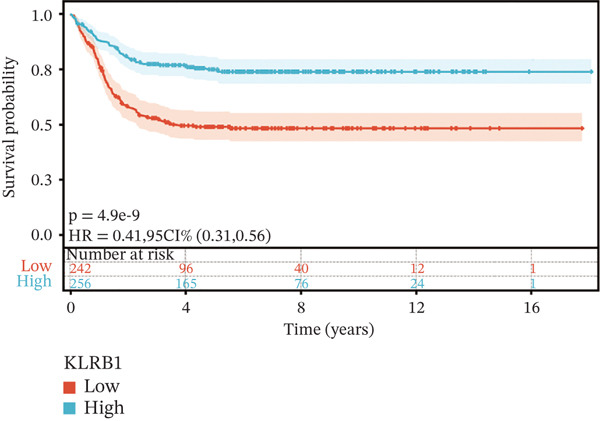
(d)

**Figure:**
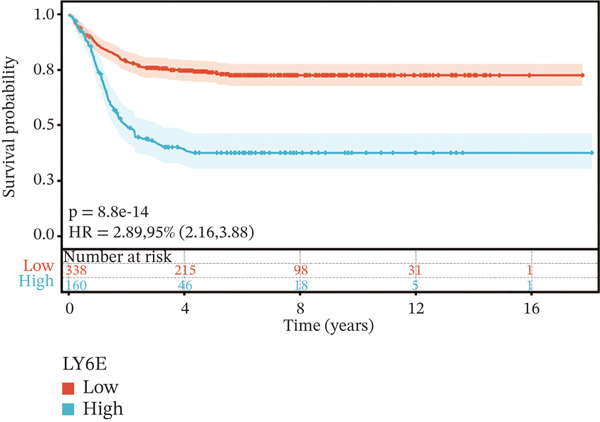
(e)

**Figure:**
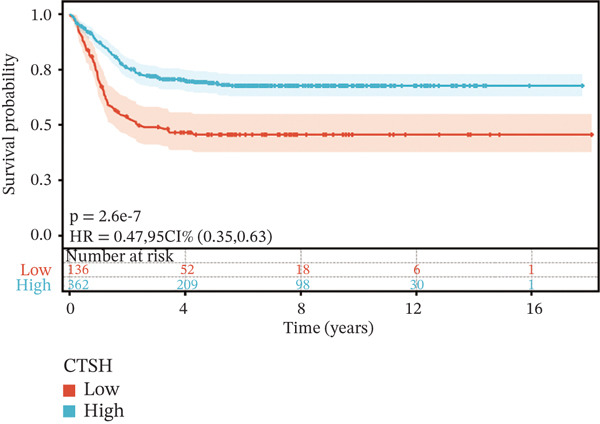
(f)

### 3.3. Prognostic Value and Predictive Accuracy of Individual Signature Genes

We then used K‐M survival analysis to assess the relationship between the six selected DEGs and prognosis, and ROC curves to evaluate the predictive accuracy of these genes. K‐M analysis revealed significant inverse correlations between CMBL/LY6E expression and survival outcomes (all *p* < 0.001), whereas PTGDS, CD3D, KLRB1, and CTSH demonstrated significant positive associations with prognosis (all *p* < 0.001) (Figures 4a, 4b, 4c, 4d, 4e, and 4f). ROC curves demonstrated that the prognostic features of CMBL, LY6E, KLRB1, CTSH, CD3D, and PTGDS performed well in predicting 1‐year, 3‐year, and 5‐year OS (Figures 5a, 5b, 5c, 5d, 5e, and 5f).

Figure 5ROC curve for survival prediction in NB. (a–f) ROC curves showing the accuracy of CD3D (a), CMBL (b), CTSH (c), KLRB1 (d), LY6E (e), and PTGDS (f) in predicting 1‐, 3‐, and 5‐year OS. The red curve indicates 1‐year AUC, the blue curve indicates 3‐year AUC, and the green curve indicates 5‐year AUC.

**Figure:**
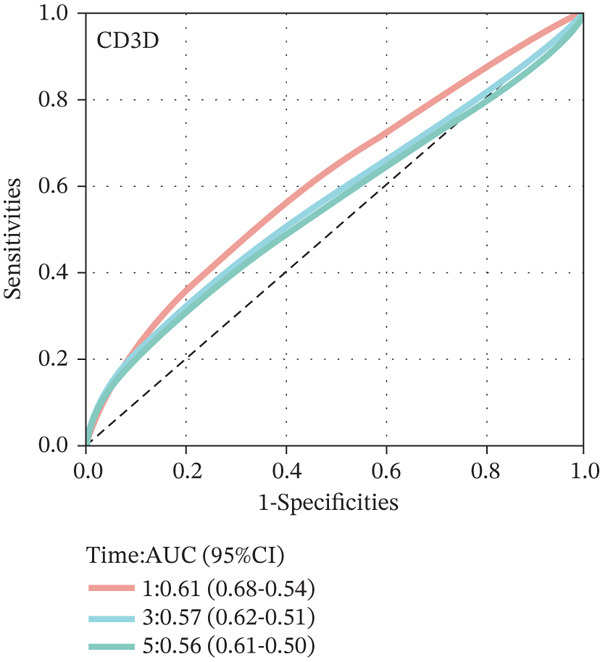
(a)

**Figure:**
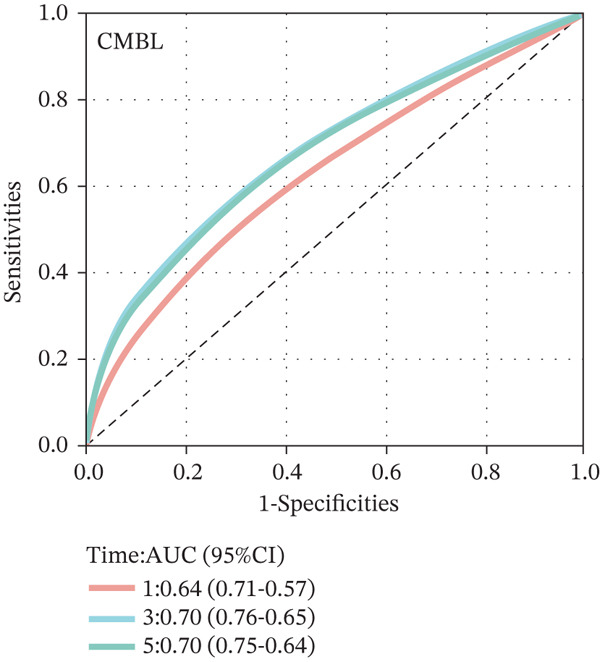
(b)

**Figure:**
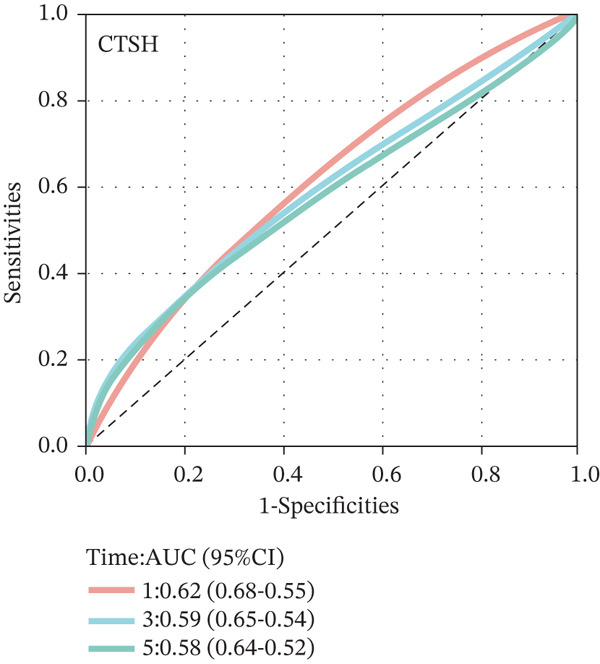
(c)

**Figure:**
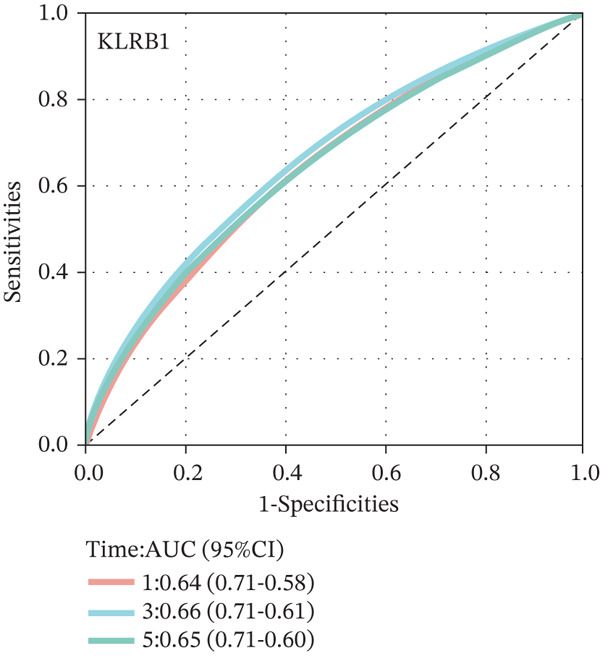
(d)

**Figure:**
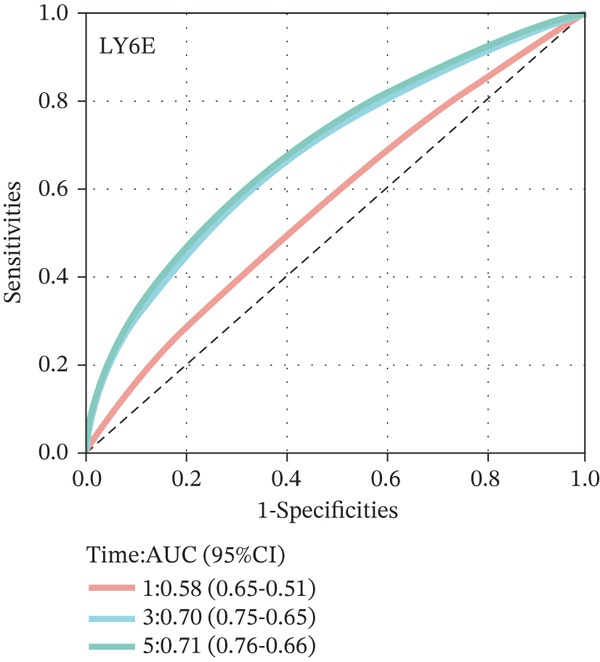
(e)

**Figure:**
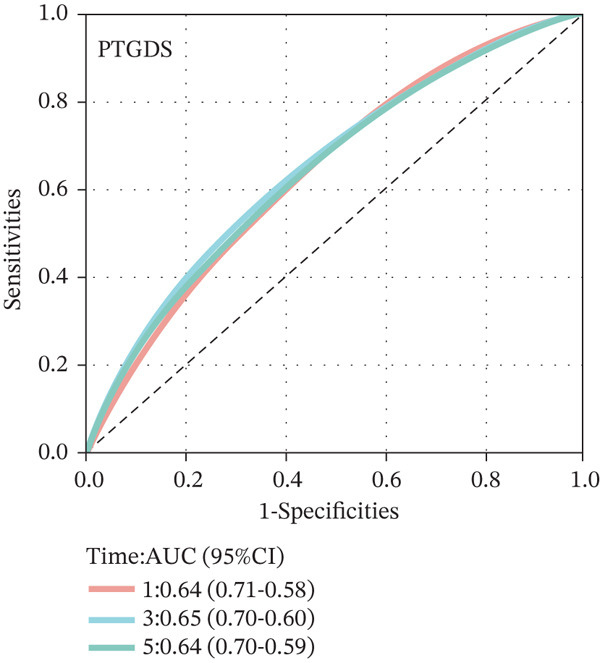
(f)

### 3.4. Clinical Relevance and Correlation Analysis of the Prognostic Signature

Subsequently, we systematically assessed the expression patterns of the risk score and the six‐gene panel (CMBL, PTGDS, CD3D, KLRB1, LY6E, and CTSH) across diverse clinical variables, including survival status, clinical risk stratification, and INSS staging. Analysis revealed statistically significant differential expression between deceased and surviving patients for all genes except CD3D (all *p* < 0.05) (Figure 6a). Marked expression differences were also observed between high‐risk and nonhigh‐risk cohorts in clinical risk stratification, with all six genes showing significant variation (all *p* < 0.0001) (Figure 6b). Furthermore, evaluation across INSS stages demonstrated that, consistent with findings in the survival status group, all genes except CD3D exhibited significant expression differences among stages (all *p* < 0.001) (Figure 6c).

Figure 6Correlation of the risk score and DEGs and several clinical parameters. (a) The expression levels of RiskScores, CMBL, PTGDS, CD3D, KLRB1, LY6E, and CTSH in both dead status group and alive status group. (b) The expression levels of RiskScores, CMBL, PTGDS, CD3D, KLRB1, LY6E, and CTSH in both high‐risk group and nonhigh risk group. (c) The expression levels of RiskScores, CMBL, PTGDS, CD3D, KLRB1, LY6E, and CTSH in each stage of INSS‐Stage.

**Figure:**
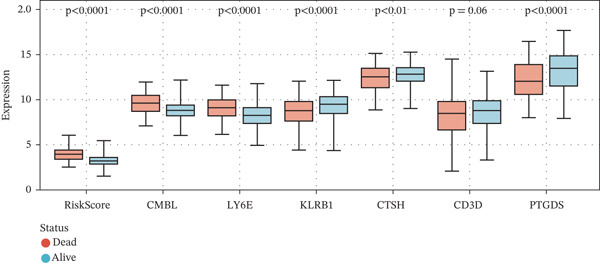
(a)

**Figure:**
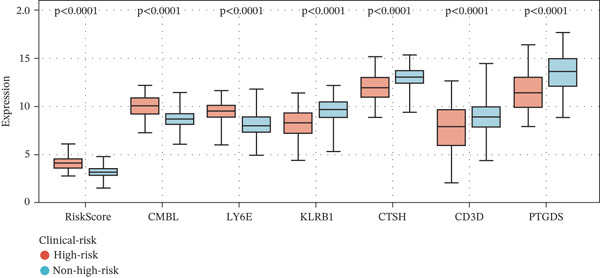
(b)

**Figure:**
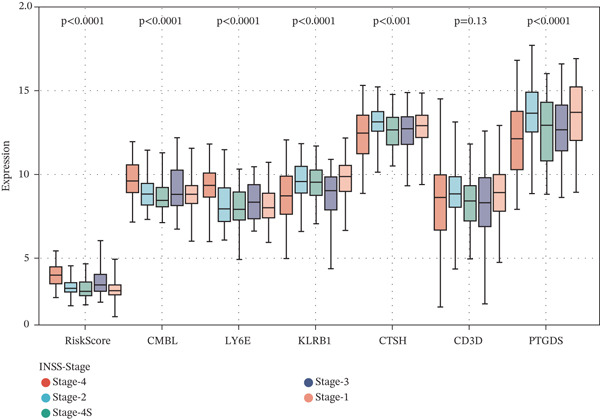
(c)

Correlation analyses further elucidated the prognostic relevance of the signature. Both the risk score and the expression levels of CMBL and LY6E showed significant inverse correlations with OS and EFS (all *p* < 0.05). In contrast, PTGDS, CD3D, KLRB1, and CTSH expression were positively associated with favorable survival outcomes (all *p* < 0.05) (Figure 7a). Additionally, gene‐to‐risk score correlation analysis confirmed that CMBL and LY6E expression correlated positively with the risk score, whereas the remaining four genes exhibited negative correlations (Figure 7b).

Figure 7Correlation analysis of six hub genes. (a) The heat map showing the correlation between gene modules and OS/EFS time. The rows represent gene modules and columns correspond to clinical traits. Each cell includes the correlation coefficient (upper number) and *p* value (lower number). (b) The correlations analysis between different gene expressions and RiskScore.

**Figure:**
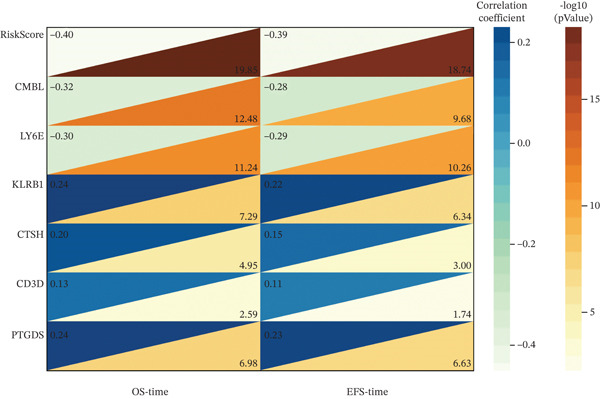
(a)

**Figure:**
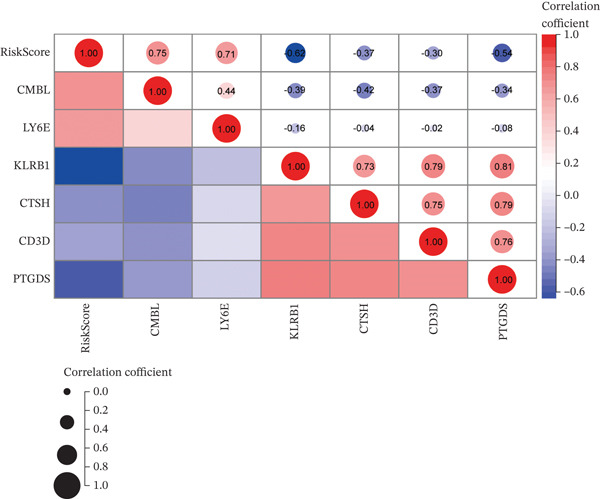
(b)

### 3.5. Correlation Analysis of the Six‐Gene Signature With Drug Sensitivity Profiles

Genomic alterations significantly influence cancer progression, therapeutic resistance, and efficacy of targeted treatments [24]. To investigate the association between DEGs and therapeutic responses, we integrated transcriptomic data and drug sensitivity profiles of cancer cell lines from the GDSC and CTRP databases. Pearson correlation analysis was systematically performed to evaluate the relationship between the six‐gene expression signature and chemosensitivity. Analysis of both the GDSC dataset and the CTRP database consistently demonstrated that elevated expression of CMBL, LY6E, CTSH, and PTGDS was positively correlated with higher IC_50_ values, indicating their association with drug resistance. Conversely, KLRB1 and CD3D expression showed negative correlations with IC_50_ values, suggesting their link to enhanced drug sensitivity (Figure 8a,b). Collectively, these findings suggest that targeted inhibition of these resistance‐associated genes (CMBL, LY6E, CTSH, and PTGDS) may represent a promising therapeutic strategy for cancer treatment, whereas promoting the expression or function of KLRB1 and CD3D could serve as an alternative approach to improve therapeutic efficacy.

Figure 8Correlational analysis of drug sensitivity in pan‐cancer. (a) The gene set drug sensitivity analysis from GDSC IC50 drug data. (b) The gene set drug sensitivity analysis from CTRP IC50 drug data. The Pearson′s correlation illustrates the relationship between six key genes′ expression and drugs′ sensitivity. Blue bubbles represented negative correlations, and red bubbles represented positive correlations; a darker color and a larger bubble denote a more obvious difference. The black outline indicates an FDR < 0.05.

**Figure:**
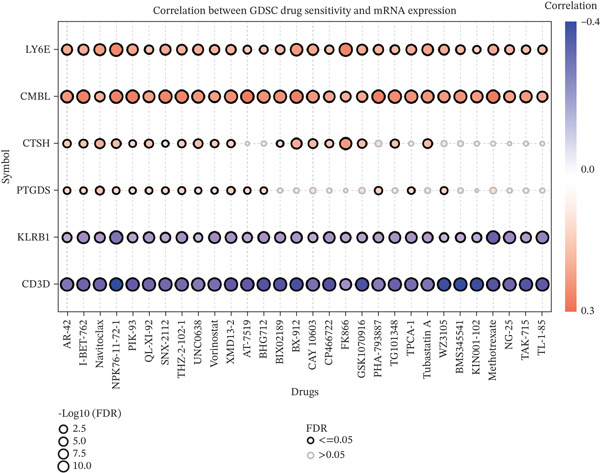
(a)

**Figure:**
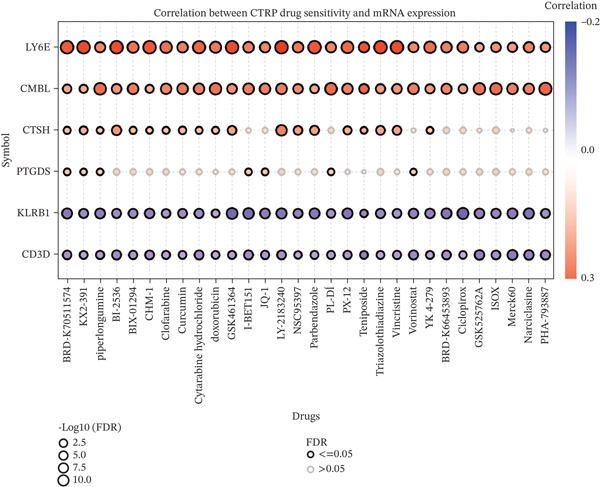
(b)

### 3.6. External Verification of the Signature

To independently validate the prognostic signature, we utilized the external GSE62564 cohort. The predictive efficacy was assessed using K‐M survival analysis and time‐dependent ROC curves. ROC analysis revealed that the signature exhibited strong predictive performance for 1, 3, and 5‐year OS with AUC values of 0.83, 0.84, and 0.85, respectively, and for EFS with corresponding AUCs of 0.64, 0.76, and 0.76 (Figure 9b,c). Moreover, LY6E alone demonstrated robust predictive accuracy for 1, 3, and 5‐year OS, achieving AUC values of 0.70, 0.71, and 0.73, respectively (Figure 9a). K‐M curves further confirmed that patients in the high‐risk group had significantly worse OS and EFS outcomes compared with the low‐risk group, a result consistent with observations from the GSE49710 dataset (Figure 9d,e). Taken together, these findings underscore LY6E as a reliable and robust prognostic biomarker for NB patient outcomes.

Figure 9The external verification of GSE62564 validation set. (a) ROC curve of LY6E in evaluating OS. (b, c) ROC curve shows the value of risk score in evaluating OS and EFS. (d, e) The OS and EFS curves of NB patients from GSE62564 red curve represents patients with low‐risk score, and the blue curve represents patients with high‐risk score.

**Figure:**
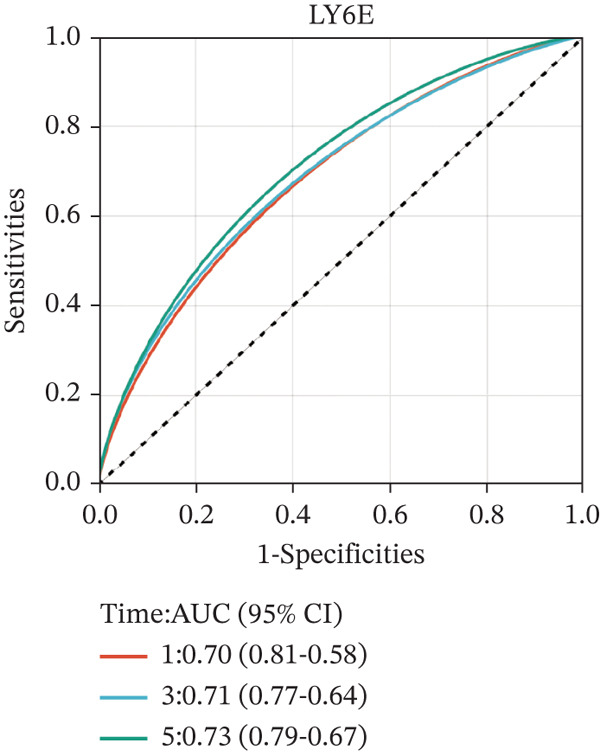
(a)

**Figure:**
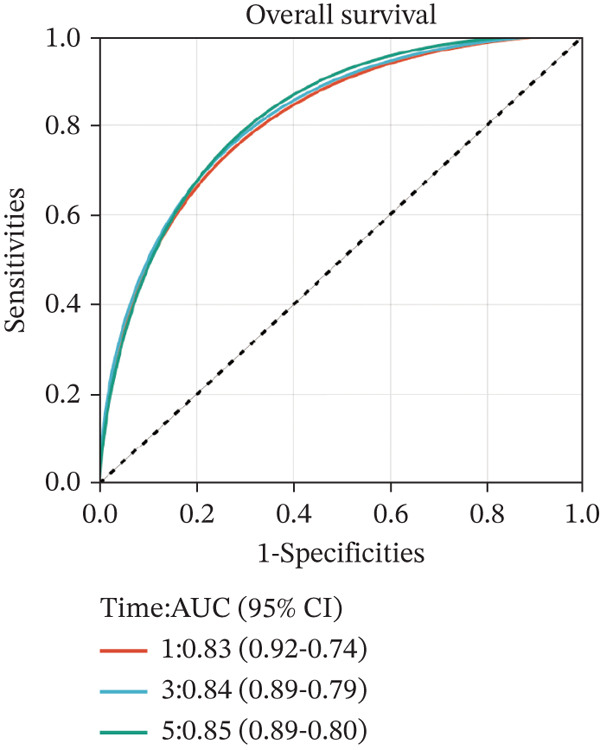
(b)

**Figure:**
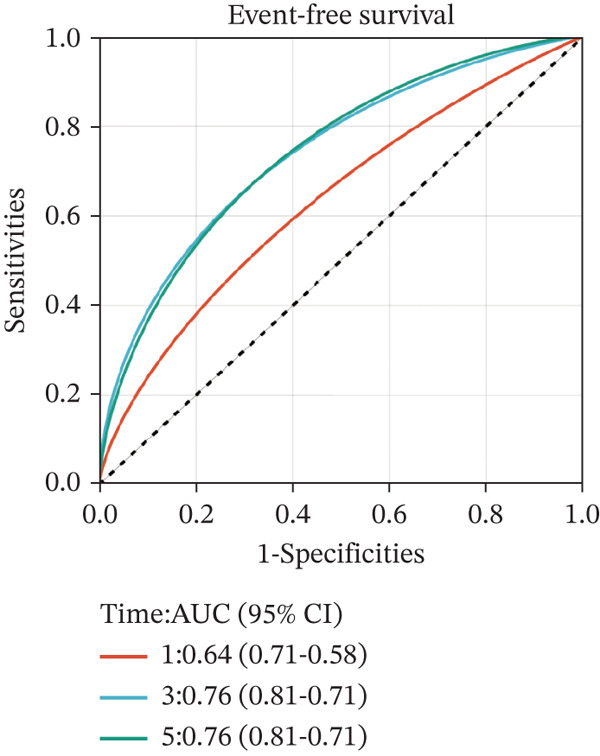
(c)

**Figure:**
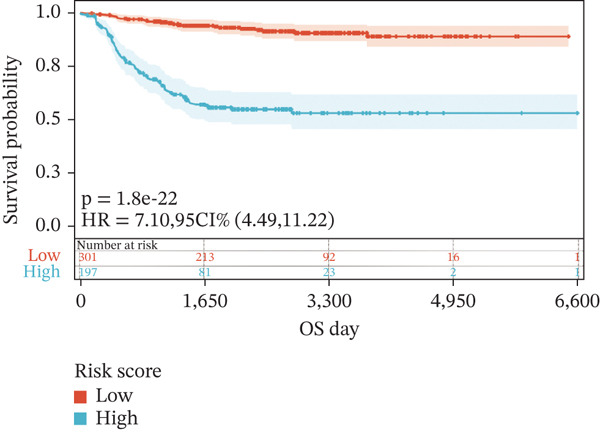
(d)

**Figure:**
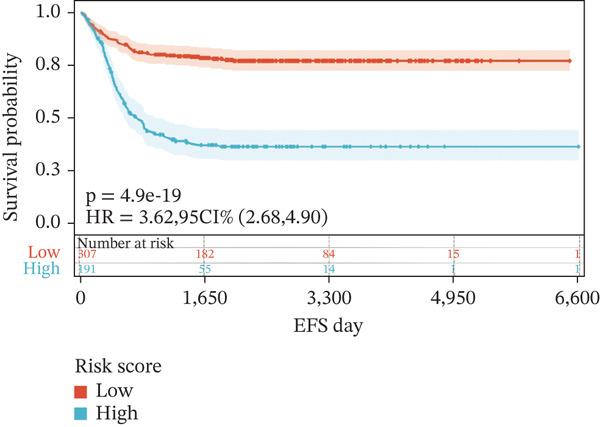
(e)

### 3.7. LY6E Regulates Proliferation and Migration of NB Cells and Promotes M2 Macrophage Polarization

To elucidate the functional role of LY6E in NB cells, initial investigations focused on phenotypic alterations following LY6E knockdown. Effective knockdown of LY6E in NB cells was achieved using siRNA (siLY6E‐1 and siLY6E‐2) (Figure 10a). CCK‐8 and EdU assays confirmed that LY6E knockdown inhibited cell viability and proliferation in both SK‐N‐BE(2) and SH‐SY5Y cells (Figure 10b,c). Meanwhile, Transwell assays demonstrated that LY6E knockdown suppressed the migratory ability of SK‐N‐BE(2) and SH‐SY5Y cells (Figure 10d). In contrast, overexpression of LY6E in NB cells exhibited an opposing trend, where elevated LY6E expression significantly enhanced cellular viability, proliferative capacity, and migratory ability (Figures 11a, 11b, 11c, and 11d).

Figure 10LY6E plays a key role in NB cell proliferation, migration, and macrophage M2 polarization. (a) The knockdown efficiency of LY6E was verified by qRT‐PCR. (b–d) LY6E knockdown in SK‐N‐BE(2) and SH‐SY5Y cells significantly reduced cell viability, proliferation capacity, and migration ability. (e) Schematic of an in vitro model in which THP‐1 macrophages were cocultured with transfected NB cells. (f) qRT‐PCR was used to measure the expression of biomarkers of M1‐like (IL‐1*β*, TNF‐*α*, CD86) and M2‐like (TGF‐*β*, CD163, CD206) macrophages in THP‐1 macrophages cocultured with NB cells. (g) ELISA was used to measure the concentrations of IL‐10 and IL‐4 in the coculture system ( ^∗^ *p* < 0.05,  ^∗∗^ *p* < 0.01,  ^∗∗∗^ *p* < 0.001).

**Figure:**
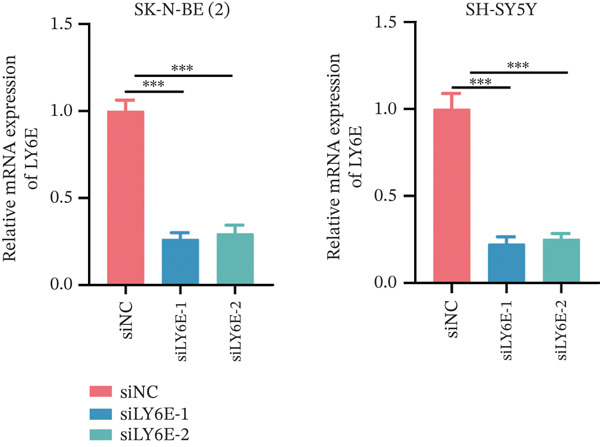
(a)

**Figure:**
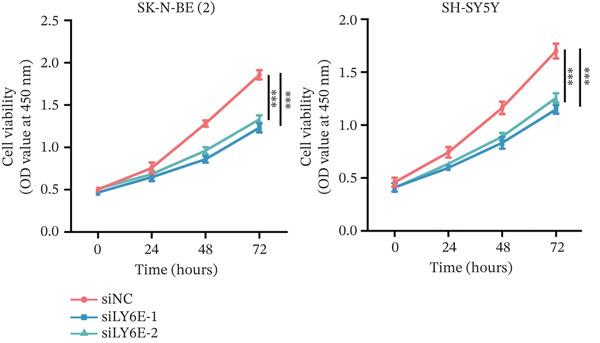
(b)

**Figure:**
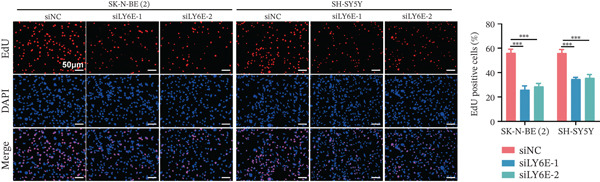
(c)

**Figure:**
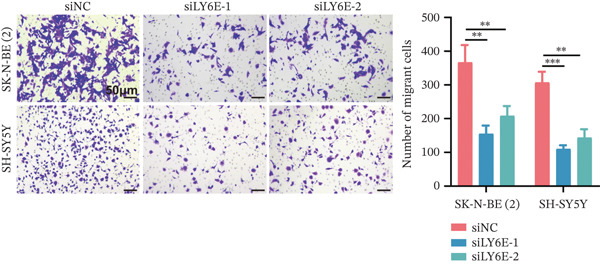
(d)

**Figure:**
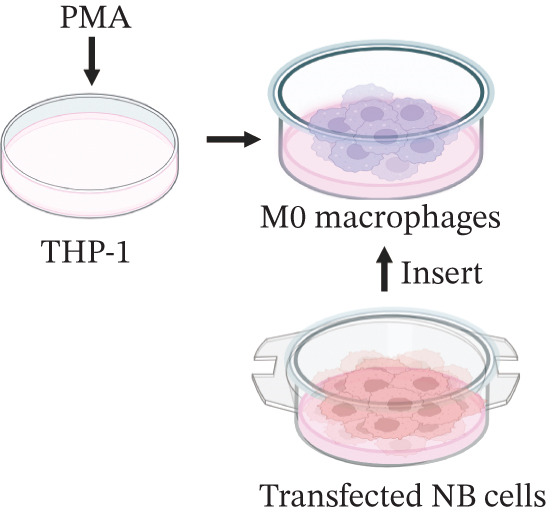
(e)

**Figure:**
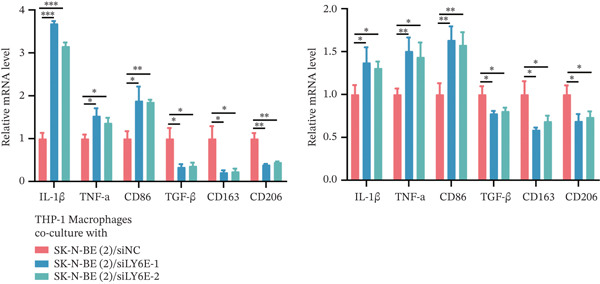
(f)

**Figure:**
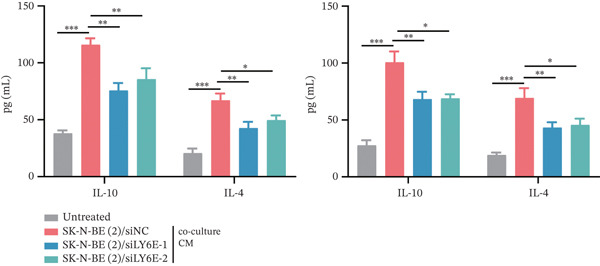
(g)

Figure 11Overexpression of LY6E promotes NB cell proliferation, migration, and macrophage M2 polarization. (a) The overexpression efficiency of LY6E was confirmed by qRT‐PCR. (b–d) Overexpression of LY6E in SK‐N‐BE(2) and SH‐SY5Y cells enhanced cellular viability, proliferative capacity, and migratory potential. (e) qRT‐PCR analysis of M1‐like (IL‐1*β*, TNF‐*α*, CD86) and M2‐like (TGF‐*β*, CD163, CD206) macrophage biomarkers in THP‐1 macrophages cocultured with LY6E‐overexpressing NB cells. (f) ELISA quantification of IL‐10 and IL‐4 concentrations in the coculture supernatant ( ^∗^ *p* < 0.05,  ^∗∗^ *p* < 0.01,  ^∗∗∗^p < 0.001).

**Figure:**
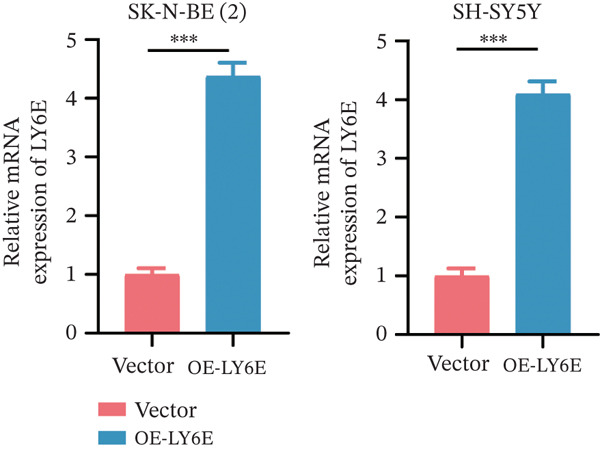
(a)

**Figure:**
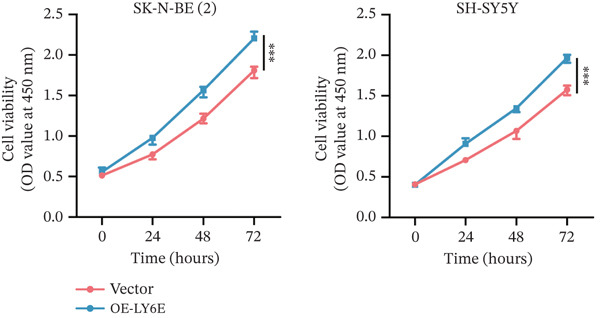
(b)

**Figure:**
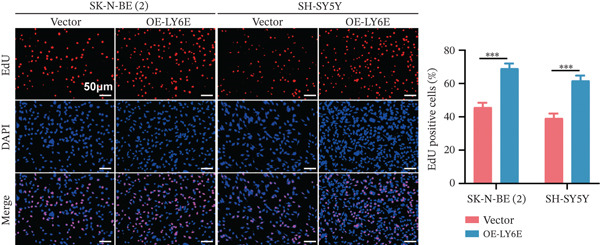
(c)

**Figure:**
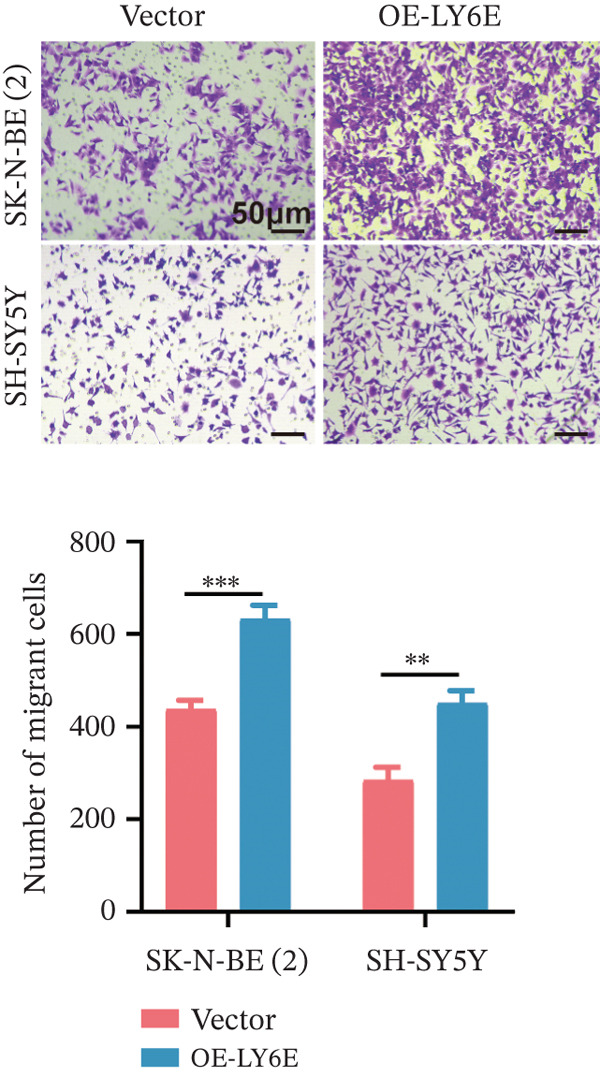
(d)

**Figure:**
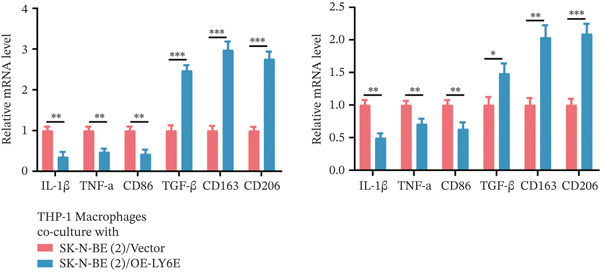
(e)

**Figure:**
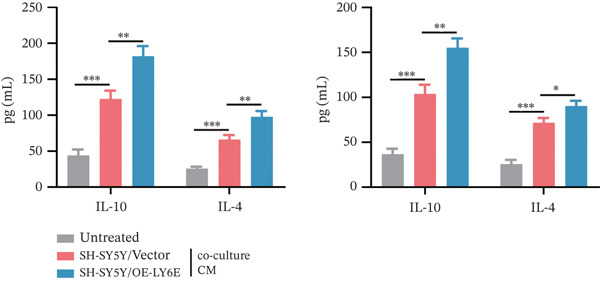
(f)

To further investigate the connection between LY6E expression in NB cells and macrophages, we cocultured M0 macrophages with NB cells in Transwell chambers after knocking down LY6E (Figure 10e). We then measured the expression levels of M1 markers and M2 markers in macrophages using qRT‐PCR. LY6E silencing markedly reduced the expression of M2 macrophage markers (TGF‐*β*, CD163, and CD206) while substantially elevating the levels of M1 phenotype markers (IL‐1*β*, TNF‐*α*, and CD86) (Figure 10f). Additionally, ELISA was conducted to assess cytokines related to the M2 phenotype. Experimental data demonstrated significantly reduced concentrations of IL‐10 and IL‐4 in the conditioned medium of THP‐1 macrophages cocultured with LY6E‐silenced tumor cells, suggesting substantial diminishment of M2‐polarized macrophages in our coculture model (Figure 10g). Conversely, in the coculture system of LY6E‐overexpressing NB cells and M0 macrophages, we found that LY6E‐overexpressing NB cells could promote the expression of TGF‐*β*, CD163, and CD206 in macrophages while inhibiting the expression of IL‐1*β*, TNF‐*α*, and CD86 (Figure 11e), meanwhile, the concentrations of IL‐10 and IL‐4 were significantly increased in this coculture system (Figure 11f). Collectively, these findings indicate that LY6E silencing exerts dual inhibitory effects on both NB cell proliferation/migration and M2 macrophage polarization.

## 4. Discussion

NB exhibits remarkable clinical heterogeneity, with disease courses ranging from spontaneous remission to progressive, treatment‐refractory malignancy. Individuals stratified into the low‐risk category typically exhibit favorable clinical outcomes and necessitate only minimal therapeutic intervention; some can even be cured through surgery alone. However, over half of NB cases are high risk, and these patients often respond poorly to treatment [25]. Approximately half of high‐risk cases exhibit resistance to first‐line treatments, resulting in poor clinical outcomes. Due to NB′s heterogeneity, some patients may develop complications such as multidrug resistance and severe toxic side effects [26].

Progress in genome‐wide association studies, transcriptomic profiling, genomic sequencing, and high‐throughput genomic methodologies have substantially advanced the comprehension of NB pathogenesis and molecular pathways, uncovering novel therapeutic targets [27, 28]. Although genetic testing is widely used in clinical oncology, the number of genes available for NB prognosis is limited, and diagnostic accuracy varies considerably. Therefore, further research is needed to identify additional factors influencing NB prognosis and to develop new treatment strategies that can improve patient outcomes [29].

The TME represents a dynamically interactive network comprising cancer‐associated fibroblasts, endothelial cells, TAMs, B cells, and T cells, all contributing significantly to oncogenic development, disease progression, and metastatic processes. Among these, TAMs and their precursors represent the largest cell population throughout tumor development [30]. TAMs predominantly enhance neoplastic proliferation, suppress immune responses, and promote vascularization, consequently accelerating oncogenesis and metastatic dissemination. Substantial evidence from multiple studies indicates that increased tumor‐associated macrophage infiltration is associated with poor prognosis across various malignancies, including lung cancer [31] and breast cancer [32]. TAMs exhibit plasticity between M1 and M2 polarization states, with the TME preferentially promoting M2 differentiation to facilitate oncogenesis, immune suppression, and metastatic dissemination in solid malignancies. This has generated significant research focus on developing immunotherapeutic strategies that specifically target TAMs, especially M2 macrophages. For instance, several drugs aim to inhibit M2 polarization. Since the STAT3 pathway is crucial for inducing M2 polarization, kinase inhibitors like sorafenib and sunitinib have been used to block this pathway [33]. The STAT6 signaling pathway further promotes M2 polarization through cytokine‐mediated activation; following IL‐4/IL‐13 binding, phosphorylated STAT6 translocates to the nucleus where it executes transcriptional regulatory functions. STAT6 inhibitors such as AS1517499 and TMC‐264 have been shown to inhibit STAT6 phosphorylation and macrophage polarization [34]. These findings highlight the importance of investigating the relationship between NB cells and immunotherapy.

We initially identified 476 DEGs associated with MYCN status (MYCN‐DEGs) by comparing NB patients with and without MNA. By integrating genes related to TAMs, we subsequently refined this list to 16 MYCN–TAM‐DEGs. From these, six genes (CMBL, LY6E, KLRB1, CTSH, CD3D, and PTGDS) were selected to develop a risk scoring model. This prognostic model demonstrated accurate prediction of OS in NB patients. Building upon prior bioinformatics analyses, the present study specifically investigated the relationship between LY6E expression and NB prognosis.

Although the Cox regression combined with the LASSO algorithm employed in this study is a well‐established approach for constructing prognostic signatures in cancer research—offering advantages of simplicity, interpretability, and clear coefficient weights for each gene—we acknowledge the potential of advanced machine learning models to further improve predictive performance. As highlighted in previous studies [35, 36], models such as the RPI‐Bind model play a vital role in investigating RNA‐protein interactions. Additionally, survival prognostic subnetwork signatures exhibit superior performance, illustrating the value of multiplatform modalities in predicting patient outcomes—specifically through networks that stratify patients based on their proliferation profiles.

KLRB1 is predominantly expressed in natural killer (NK) cells, *γδ* T cells, and CD4^+^ T cell subsets [37], and its high expression correlates with favorable prognosis in NB (HR = 0.77, *p* = 9.8*e* − 3). As an activating receptor, KLRB1 can enhance the cytotoxicity of NK cells and the secretion of IFN‐*γ* and TNF‐*α* via recognition of ligands on tumor cells. Therefore, in NB, KLRB1^+^ immune cells may infiltrate the TME to exert tumoricidal effects and suppress M2 macrophage polarization through proinflammatory cytokines. In addition, accumulating evidence has indicated that CD3D expression is significantly associated with multiple malignancies, including colorectal cancer, highlighting its potential as a prognostic biomarker. Meanwhile, CD3D encodes the T cell receptor *δ* chain, which is essential for T cell activation [38, 39].

Previous studies have shown that the LY6 family is located on Chromosomes 6, 8, 11, and 19 and includes LY6D, LY6E, LY6H and LY6K, which are widely expressed in human lymphoid cells and can modulate immune cell activation [18, 40]. Moreover, a substantial body of research has demonstrated that the LY6 family is implicated in the progression of aggressive and treatment‐refractory cancers. Specifically, the mRNA expression levels of several LY6 family genes are markedly elevated in various malignancies, including lung, brain, breast, and head and neck cancers, compared with normal tissues. LY6E is a glycosylphosphatidylinositol‐anchored cell surface protein that associates with the cell membrane through its conserved cysteine residues, thereby facilitating signal transduction and cellular activation [41]. Furthermore, LY6E has been shown to modulate cellular proliferation, oncogenic processes, differentiation, and immune regulation. Significantly, LY6E has been recognized as a frequently expressed surface antigen in gastric carcinoma, pancreatic cancer, and triple‐negative breast cancer—a subtype defined by the lack of estrogen receptor, progesterone receptor, and human epidermal growth factor Receptor 2 expression [16]. The expression of LY6E in NB has not been documented, particularly regarding its function in the regulation of TAMs within the TME. Consequently, further investigation is required to clarify the potential interplay between LY6E and TAMs in

In subsequent experimental validation of our preliminary findings, we initially investigated the functional role of LY6E in two NB cell lines, SK‐N‐BE(2) and SH‐SY5Y. The results demonstrated that LY6E silencing in NB cells significantly inhibited neoplastic proliferation and migration. Furthermore, upon coculturing LY6E‐knockdown NB cells with THP‐1‐derived macrophages, analysis of key M1 and M2 macrophage polarization markers revealed that LY6E knockdown notably inhibited M2‐type macrophage polarization. Additionally, it reduced concentrations of IL‐4 and IL‐10 in macrophage‐conditioned medium.

While our six‐gene signature effectively stratifies NB patients into high‐ and low‐risk groups with significantly distinct survival outcomes (*p* < 0.001) and exhibits robust predictive performance in the external validation cohort, we acknowledge its modest performance in the discovery cohort, where the 5‐year OS AUC falls below 0.6. This relatively low AUC in the discovery cohort may be attributed to several factors: first, the inherent heterogeneity of NB, as this cohort includes patients with diverse clinical characteristics (e.g., age, International Neuroblastoma Staging System [INSS] stage, and genetic backgrounds) that could introduce confounding variables; second, the model was constructed solely based on transcriptomic data of six MYCN‐TAM‐related genes, without integrating other multiomics data or clinical variables (e.g., age and MNA status) known to influence NB prognosis. Collectively, these factors may limit the predictive precision of the model in the discovery cohort.

This study represents the pioneering exploration of NB‐TAM interactions within the immune microenvironment. The experimental findings presented herein are expected to advance the comprehension of the molecular mechanisms underlying NB and to contribute to the refinement of diagnostic and prognostic approaches. While in the context of our study, the six‐gene signature constructed via LASSO‐Cox regression achieves satisfactory predictive accuracy (AUC values of 0.70, 0.64, and 0.57 for 1‐, 3‐, and 5‐year OS in the training cohort, and 0.83, 0.84, and 0.85 in the validation cohort), indicating its clinical applicability. However, future studies could explore advanced models to integrate additional multiomics data or clinical variables with the six‐gene signature, potentially further enhancing prognostic precision. It is worth noting that advanced models often face challenges of reduced interpretability and increased computational complexity, which may limit their clinical translation. Thus, a balance between predictive performance and practical utility should be considered when selecting modeling approaches. Our current LASSO‐Cox model, with its clear risk score formula and biological interpretability, provides a straightforward tool for clinical risk stratification of NB patients, whereas advanced models offer promising directions for optimizing prognostic signatures in future research. Moreover, LY6E exhibited notable prognostic significance in this study. Our study utilized GEO datasets for gene signature construction and external validation. Although these datasets include a large number of NB patient samples and cover diverse clinical characteristics, they still have inherent limitations: First, public transcriptomic data may be affected by technical variations and batch effects, which could potentially influence the reliability of DEG identification and prognostic model construction. Second, the clinical metadata in GEO datasets is relatively limited, preventing us from analyzing the association between the six‐gene signature and treatment response in specific subgroups. To address these limitations, future studies should validate the six‐gene signature and LY6E′s prognostic value using multicenter, prospective clinical samples from diverse ethnic backgrounds. Collecting detailed clinical information will enable stratification analysis to determine the signature′s utility in different treatment contexts. Additionally, integrating multiomics data from self‐collected samples will help refine the prognostic model and improve its clinical applicability. Moreover, our functional experiments were limited to in vitro cell culture and Transwell coculture systems. Future studies should establish in vivo NB models to validate LY6E′s biological function, such as xenograft models can be constructed by implanting LY6E‐silenced or overexpressed NB cells into nude mice, to observe the effects of LY6E on tumor growth rate, volume. Second, humanized mouse models can be used to investigate the impact of LY6E knockdown on the infiltration and polarization of human TAMs in the TME. These in vivo experiments will provide critical preclinical evidence for LY6E as a potential therapeutic target. Furthermore, the present study primarily focuses on elucidating the functional phenotype of LY6E in regulating M2 macrophage polarization, whereas an in‐depth characterization of its downstream signaling mechanisms remains unexplored. Nevertheless, based on the data generated in this study (e.g., Figure 10g) combined with findings from existing literature, we preliminarily hypothesize that LY6E may mediate this process by modulating the secretion of specific cytokines (e.g., IL‐10 and IL‐4) in tumor cells. To clarify the exact underlying mechanism, systematic validation via additional in vivo and in vitro experiments will be conducted in our future work.

Its efficacy in disease risk stratification necessitates validation through extensive clinical cohort studies. Future research endeavors will prioritize the clinical applicability of LY6E in NB management, alongside in‐depth mechanistic analyzes aimed at identifying prospective therapeutic targets.

## 5. Conclusion

Analysis of the GSE49710 dataset revealed LY6E as a prognostic biomarker significantly associated with survival outcomes in NB. Furthermore, examination of the correlations among LY6E expression, OS rates, and chemosensitivity indicates this gene′s pivotal role in NB progression, treatment resistance, and unfavorable clinical outcomes. In summary, these results establish LY6E as a clinically significant prognostic biomarker and a potential molecular target for precision medicine approaches in NB.

## Author Contributions

**Lijuan Li:** writing—review and editing, writing—original draft, software, methodology, conceptualization. **Yu Zeng:** writing—review and editing, writing—original draft, supervision, project administration. **Qinfen Zhang:** visualization, investigation. **Gaojian Zhuang:** software, methodology. **Yu Liu:** Data curation. **Xuan Wang:** writing—review and editing, resources, supervision. **Yuqi Wang:** writing—review and editing, supervision, project administration. **Lijuan Li** and **Yu Zeng** contributed equally to this study.

## Funding

This study was supported by Tianjin Key Medical Discipline Construction Project (TJYXZDXK‐3‐003A).

## Disclosure

All authors read and approved the final manuscript for publication.

## Ethics Statement

Given that our work only involved cell experiments, therefore ethical approval was not required for this study according to the local legislation and institutional requirements.

## Conflicts of Interest

The authors declare no conflicts of interest.

## Supporting Information

Additional supporting information can be found online in the Supporting Information section.

## Supporting information


**Supporting Information 1** Figure S1: The ROC curve showed the accuracy of six DEGs in our prognostic signature in predicting 1‐, 3‐, and 5‐year EFS.

**Supporting Information 2** Table S1: Sequences of siRNA and plasmids.

**Supporting Information 3** Table S2: Primers for RT‐qPCR.

## Data Availability

The data that support the findings of this study are available on request from the corresponding authors. The data are not publicly available due to privacy or ethical restrictions.
