# Supplementary figures and images for: Targeting LY6E Inhibits Neuroblastoma Progression and Suppresses M2 Macrophage Polarization
Source: Hum Mutat. 2026 Apr 15;2026:3003097. doi: 10.1155/humu/3003097 (PMC13080879; doi:10.1155/humu/3003097)

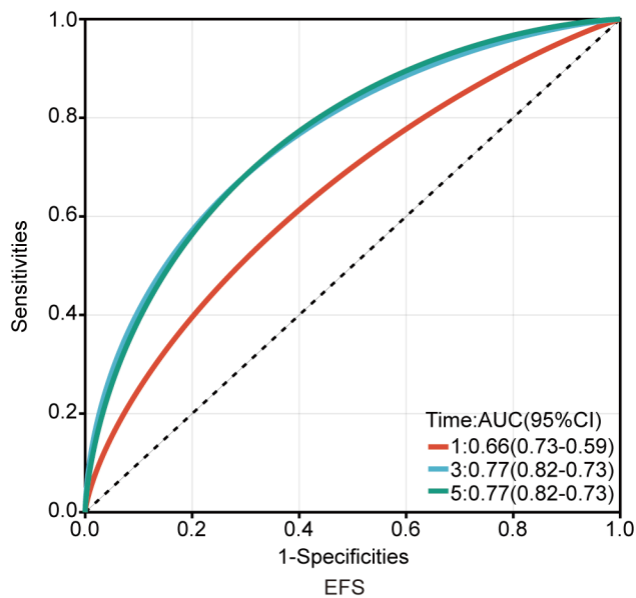

Supplement: Supplementary file 1 — Supporting Information 1 Figure S1: The ROC curve showed the accuracy of six DEGs in our prognostic signature in predicting 1‐, 3‐, and 5‐year EFS. [file HUMU-2026-3003097-s001.pdf]
