# Supplementary material for: Targeting LY6E Inhibits Neuroblastoma Progression and Suppresses M2 Macrophage Polarization
Source: Hum Mutat. 2026 Apr 15;2026:3003097. doi: 10.1155/humu/3003097 (PMC13080879; doi:10.1155/humu/3003097)
Supplement: Supplementary file 2 — Supporting Information 2 Table S1: Sequences of siRNA and plasmids. [file HUMU-2026-3003097-s003.docx]

**Supplementary Table**

**1. Primers for RT-qPCR**

| **Gene name** | **Forward Primer (5'-3')** | **Reverse Primer (5'-3')** |
| --- | --- | --- |
| β-Actin | ACAGAGCCTCGCCTTTGCCG | TTGCACATGCCGGAGCCGTT |
| LY6E | GACCAGGACAACTACTGCGTGA | AAGCCACACCAACATTGACGCC |
| IL-1β | ATGATGGCTTATTACAGTGGCAA | GTCGGAGATTCGTAGCTGGA |
| TNF-α | CTCTTCTGCCTGCTGCACTTTG | ATGGGCTACAGGCTTGTCACTC |
| TGF-β | TACCTGAACCCGTGTTGCTCTC | GTTGCTGAGGTATCGCCAGGAA |
| CD86 | CTGCTCATCTATACACGGTTACC | GGAAACGTCGTACAGTTCTGTG |
| CD163 | CCAGAAGGAACTTGTAGCCACAG | CAGGCACCAAGCGTTTTGAGCT |
| CD206 | AGCCAACACCAGCTCCTCAAGA | CAAAACGCTCGCGCATTGTCCA |

**2. Sequences of siRNA and overexpression plasmids**

| **siRNA** | **Sense Sequence** | **Anti-Sense Sequence** |
| --- | --- | --- |
| siLY6E-1 | CAUUGGGAAUCUCGUGACA  (dT)(dT) | UGUCACGAGAUUCCCAAUG  (dT)(dT) |
| siLY6E-2 | GUCUUACGGUCCAACAUCA  (dT)(dT) | UGAUGUUGGACCGUAAGAC  (dT)(dT) |

The siRNA for the Negative Control (NC) group, along with the LY6E overexpression plasmid and its control, were commercially sourced from Tsingke.
